# Prevalence and predictors of poor heart failure treatment outcomes in Ethiopia: a systematic review and meta-analysis

**DOI:** 10.3389/fcvm.2024.1434265

**Published:** 2024-12-24

**Authors:** Firomsa Bekele, Lalise Tafese, Ginenus Fekadu, Geleta Nenko Dube, Dinka Dugassa, Dagim Samuel

**Affiliations:** ^1^ Department of Pharmacy, Institute of Health Science, Wallaga University, Nekemte, Ethiopia; ^2^ Department of Health Informatics, College of Health Science, Mattu University, Mattu, Ethiopia; ^3^ Department of Infectious Diseases and Public Health, Jockey Club College of Veterinary Medicine and Life Sciences, City University of Hong Kong, Hong Kong, Hong Kong SAR, China

**Keywords:** treatment outcome, associated factors, Ethiopia, heart failure, prevalence

## Abstract

**Background:**

Heart failure (HF) is a leading cause of morbidity and mortality worldwide. Various factors can exacerbate disease progression in patients with HF and negatively impact treatment outcomes. This study aims to evaluate the pooled prevalence and contributing factors associated with poor heart failure treatment outcomes in Ethiopia.

**Methods:**

A systematic review and meta-analysis were conducted using five databases: Google Scholar, ScienceDirect, Hinari, PubMed, and Scopus. In total, 12 studies met the eligibility criteria for inclusion in this analysis. The review followed the Preferred Reporting Items for Systematic Reviews and Meta-Analyses 2020 guidelines. Data extraction was performed using a Microsoft Excel spreadsheet, and statistical analysis was conducted with STATA 14. The Joanna Briggs Institute Meta-analysis of Statistics Assessment and Review Instrument was utilized for quality assessment. Heterogeneity among the studies was evaluated using the *I*^2^ statistic and the Cochrane *Q* test. Publication bias was assessed using Begg's test, Egger's weighted regression, and funnel plots.

**Results:**

The pooled prevalence of poor HF treatment outcomes was found to be 16.67% [95% confidence interval (CI): 10.67–22.67]. No significant heterogeneity was observed across the included studies (*I*^2^ = 0.0%, *p* = 0.962). Significant predictors of a poor treatment outcome were smoking cigarettes [adjusted odds ratio (AOR) = 10.74; 95% CI: 3.24–35.63] and medication-related problems (AOR = 3.99; 95% CI: 1.90–8.37).

**Conclusion:**

The prevalence of poor HF treatment outcomes in Ethiopia was found to be high. Smoking cigarettes and medication-related problems are significant predictors of these adverse outcomes. Comprehensive health education and improved clinical pharmacy services are essential for addressing these issues.

**Systematic Review Registration:**

https://www.crd.york.ac.uk/prospero/display_record.php?ID=CRD42023437397, PROSPERO (CRD42023437397).

## Background

Heart failure (HF) is a clinical syndrome characterized by the heart's diminished capacity to pump and/or fill with blood (1). It occurs when the body's metabolic needs are not met by the heart's ability to pump a sufficient volume of blood (2). Clinical signs of HF include dyspnea, fatigue, exercise intolerance, and fluid retention (3).

Globally, HF impacted over 64.3 million people in 2017 (4). The incidence of heart failure rises sharply with age; it affects 1%–2% of individuals in the 40–59 years age group and up to 12% of those older than 80 years. Heart failure is a leading cause of morbidity and mortality worldwide (5–7). Within a year of diagnosis, 30%–40% of patients succumb to the condition globally (8). Hospitalization for HF is associated with a high risk of mortality, both in the short and long term (9). The prognosis for HF remains dire, with a 50% 5-year mortality risk upon diagnosis—higher than that of several cancer types (10).

Many variables contribute to an accelerated progression of the disease and diminished treatment responsiveness in patients with HF (6). Approximately two out of every three Asian patients with widespread HF have at least two comorbidities. Poorer outcomes are linked to multimorbidity patterns in patients with HF (10).

Most patients, regardless of symptom severity, require lifelong optimal medical treatment, which includes angiotensin-converting enzyme inhibitors (ACEIs), beta-blockers (BB), and mineralocorticoid receptor antagonists (MRAs) to reduce hospitalization and mortality (11). Despite advancements in pharmaceutical therapy, HF morbidity and mortality remain high. Therefore, non-pharmacological management, primarily focusing on self-care, deserves increased attention (12).

Management of HF encompasses both lifestyle modifications and pharmacological interventions. Hospitalization rates within 6 months of discharge between 30% and 40%, and morbidity and mortality rates remain elevated, with quality of life still low despite breakthroughs in prevention and therapy (8, 11, 13–15). It has been observed that older, male, and African-American patients experience higher mortality rates (9, 14). Urgent interventions should be prioritized to address the underlying causes of HF, halt its progression, and redesign healthcare delivery, infrastructure, and treatment options (16).

In Ethiopia, heart failure-related mortality rates range from 10.2% to 32.6% (6, 7, 11, 13–17). Several risk factors, including comorbidity, advanced HF class, lower left ventricular ejection fraction (LVEF), male sex, and adverse medication events, have been associated with poor treatment outcomes (6, 13, 17, 18).

Furthermore, in Ethiopia, the management and outcomes of HF are particularly concerning due to limited healthcare resources and access to specialized care. The healthcare system often faces challenges such as inadequate diagnostic facilities, a shortage of trained healthcare professionals, and limited availability of essential medications. These factors contribute to suboptimal treatment adherence and poorer health outcomes for patients with HF. Furthermore, cultural perceptions and stigma associated with chronic illnesses may deter individuals from seeking timely medical care, exacerbating the burden of HF in the population.

This study aims to summarize recent findings on the prevalence of, and factors related to, poor treatment outcomes to inform appropriate interventions. Despite inconsistent reports on the prevalence and predictors of HF treatment outcomes in Ethiopia, no systematic review and meta-analysis has been conducted.

## Methods

### Search strategy

This systematic review and meta-analysis followed the Preferred Reporting Items for Systematic Reviews and Meta-Analyses (PRISMA) 2020 guidelines (19). Five databases—PubMed, Science Direct, Scopus, HINARI, and Google Scholar—were utilized for the literature search. The review was conducted from 12 November to 11 December 2023, with the final search conducted on 8 December 2023. The search strategy employed the following MeSH terms: [(Burden) OR (Mortality) AND (Predictors) AND (HF) AND (Ethiopia)]. The review protocol was registered with PROSPERO (CRD42023437397).

### Data collection process, items, and extraction

Two authors (FB and LT) were responsible for collecting relevant literature. Reference management software (EndNote X7.2) was used to combine the results and eliminate duplicates. Three data extractors (GD, GF, and DS) utilized a standardized data extraction checklist on Microsoft Excel. For the primary outcome (burden), the checklist included the author's name, publication year, study area, study design, sample size, and the number of individuals with the outcome. For the secondary outcome (predictors), data were extracted in the form of two-by-two tables to calculate the log odds ratio (OR) based on the original research results. Any disagreements between the two independent reviewers were resolved through discussion, with a third reviewer (DD) involved if consensus was not reached.

### Eligibility criteria

Studies were included if they reported primary outcomes and full texts were accessible, specifically focusing on the burden and determinants of poor HF treatment outcomes in Ethiopia. Excluded articles included systematic reviews, meta-analyses, preprints, brief communications, letters to the editor, and articles with unclear primary outcomes. Qualitative and non-observational studies were excluded; however, cross-sectional studies were retained. The Condition, Context, and Population (CoCoPop) framework was employed to assess study eligibility: the population consisted of patients with HF, the condition was defined as treatment outcomes for HF, and the setting was Ethiopia.

### Outcome measurement

Two primary outcomes were assessed. The burden of poor HF treatment outcomes was defined as the total number of patients with poor outcomes divided by the total number of HF patients, multiplied by 100. The second outcome involved determining factors associated with poor treatment outcomes, using ORs and binary outcomes from the included studies. Poor outcomes were operationally defined as hospital mortality related to HF, self-discharge, and HF sequelae (17).

### Quality assessment

Quality assessment was conducted using the Joanna Briggs Institute Meta-Analysis of Statistics Assessment and Review Instrument (JBI-MAStARI) (20) which is tailored for cross-sectional studies and has more univocal items for observational studies. This tool can be used without modifying its domains, unlike other tools such as the Newcastle–Ottawa Scale. The Newcastle–Ottawa Scale tool is only validated for case–control and longitudinal studies. The quality of the included studies was assessed based on several criteria: clarity in the definition of inclusion criteria for the sample, a detailed description of the study subjects and setting, the validity and reliability of exposure measurement, use of objective, standard criteria to measure the condition, identification of confounding factors, articulation of strategies to address these confounding factors, the validity and reliability of the outcome measurement, and the appropriateness of the statistical analysis utilized. Two authors (FB and GD) conducted the quality assessment and any disagreements were resolved by a third reviewer (DS). Articles were classified as high quality if the score was greater than 80%, moderate if between 65% and 80%, and low if below 65%.

### Data analysis and synthesis

Data were exported to STATA V. 14 to determine the pooled effect size with 95% confidence intervals (CIs). The Cochran *Q* test (chi-squared statistic) and *I*^2^ statistic were computed to assess heterogeneity among the included studies. A *p*-value of <0.05 indicated statistically significant heterogeneity. *I*^2^ values were interpreted as follows: 0% (no heterogeneity), 25% (minimal), 50% (moderate), and 75% (high). A funnel plot was employed to assess publication bias, with asymmetry indicating potential bias. In addition, Egger's weighted regression and Begg's test were conducted to evaluate publication bias, with statistical significance set at *p* < 0.05.

## Results

**Figure 1:**
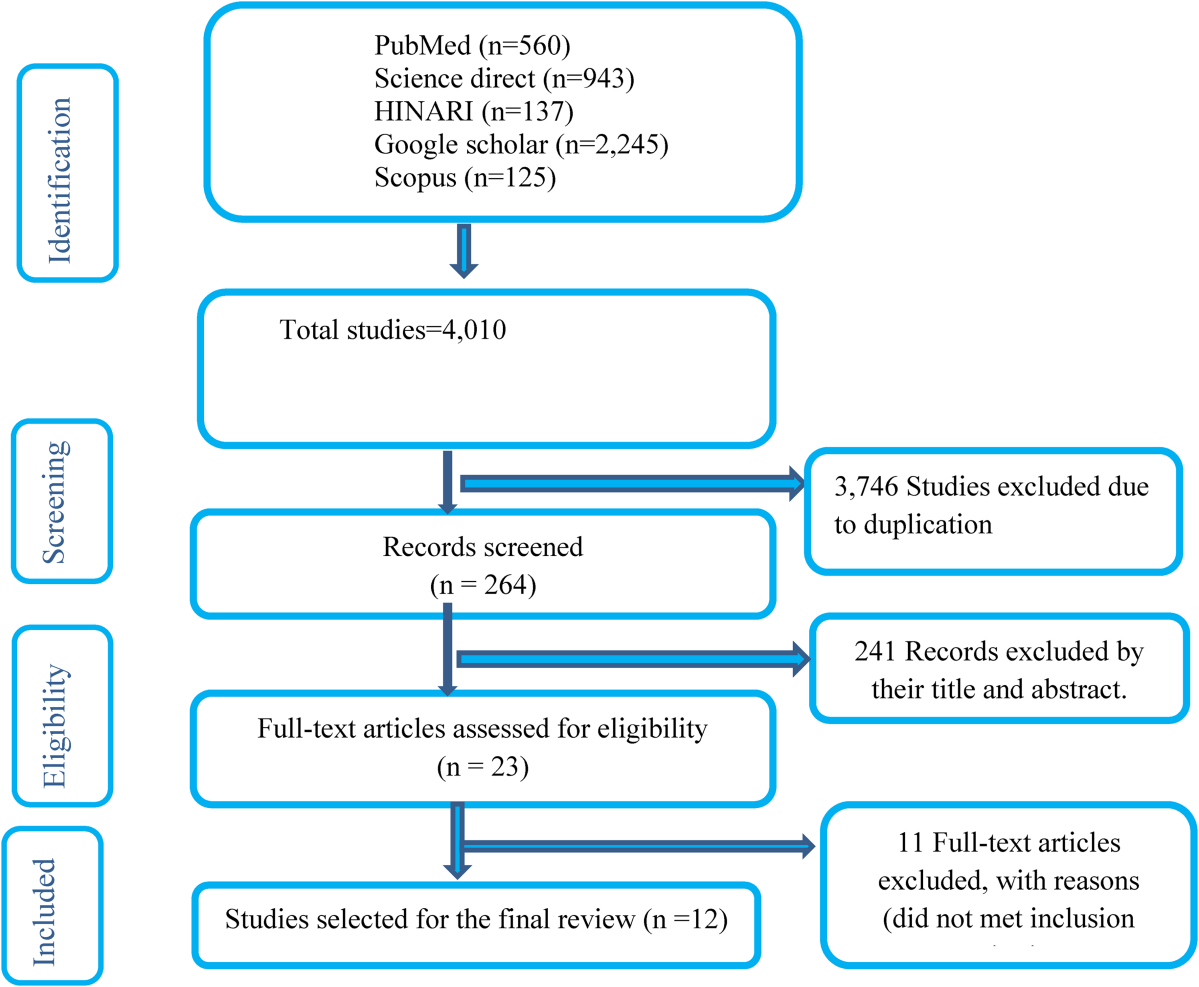
Flow chart of the systematic research and study selection process.

A total of 4,010 articles were identified from initial searches conducted in PubMed, ScienceDirect, Hinari, Scopus, and Google Scholar. After removing 3,746 duplicate articles, 241 additional articles were excluded based on title and abstract screening. Consequently, 23 articles underwent full-text review, and ultimately, 12 articles were selected for inclusion in this review (Figure 1).

### Characteristics of the included studies

The articles included in our systematic review and meta-analysis were observational studies. The majority of participants were female, as reported in seven of the articles (6, 7, 13, 17, 21–23). The total sample size was 3,002 patients with HF, with individual study sample sizes ranging from 96 to 496. Regarding study settings, five articles originated from Amhara (6, 13, 21–23) and five from Oromia (7, 11, 24–26), while two articles were based in Addis Ababa (17, 27) (Table 1).

**Table 1:** Summary of included studies on the burden and associated factors of poor treatment outcomes among patients with HF in Ethiopia, 2024.

Authors	Year of publication	Region	Study design	Sample size, sex (female), poor outcome (95%CI)
Tirfe et al. (17)	2020	Addis Ababa	Prospective observational	169, 54.4%, 17.2% (11.8–23.71)
Assefa et al. (11)	2023	Oromia	Prospective observational	242, 47.9%, 24.0% (18.73–29.85)
Tigabe et al. (13)	2021	Amhara	Cross-sectional	226, 59.3%, 10.6% (7.1–14.7)
Bogale and Aderaw et al. (21)	2021	Amhara	Cross-sectional	96, 51%, 22.9% (14.95–32.61)
Woldeyes et al. (27)	2020	Addis Ababa	Cross-sectional	496, 42.2%, 24.4% (20.68–28.42)
Moyehodie et al. (28)	2022	Amhara	Retrospective cohort	285, 52.6%, 32.6% (27.22–38.41)
Meshesha et al. (7)	2021	Oromia	Cross-sectional	252, 54.8%, 21.29% (16.53–27.02)
Hassen et al. (22)	2019	Amhara	Cross-sectional	300, 58%, 11% (7.69–15.10)
Beri et al. (24)	2023	Oromia	Prospective observational	283, 47.7%, 10.2% (6.97–14.39)
Kebede et al. (23)	2021	Amhara	Prospective cohort	228, 55.3%, 12.7% (8.69–17.75)
Bekele et al. (25)	2024	Oromia	Prospective observational	241, 42.7%, 14.9% (10.69–20.08)
Tegene et al. (26)	2022	Oromia	Prospective cohort	184, 46.7%, 20.1% (21.88–35.35)

CI, confidence interval.

### Quality assessment of included studies

In our systematic review and meta-analysis, eight articles (7, 11, 13, 17, 23–25, 27) were classified as high quality, while the remaining four articles (6, 21, 22, 26) were categorized as moderate quality according to the JBI-MAStARI.

### The magnitude of poor heart failure treatment outcome

**Figure 2:**
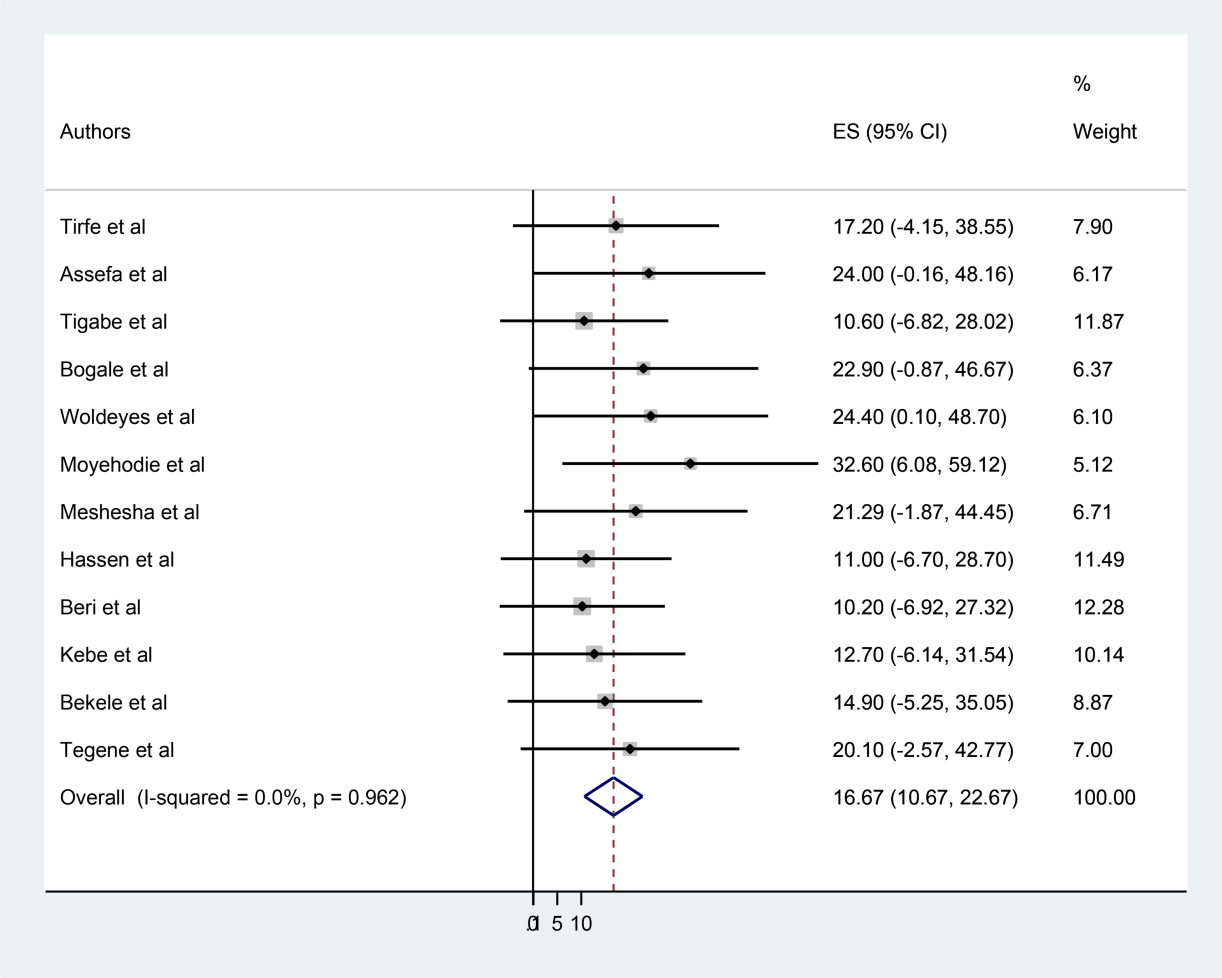
Forest plot of the pooled prevalence of poor heart failure treatment outcomes in Ethiopia, 2024.

The pooled magnitude of poor heart failure treatment outcomes was found to be 16.67% (95% CI: 10.67–22.67). No heterogeneity was observed across the included studies (*I*^2^ = 0.0%, *p* = 0.962). This lack of heterogeneity suggests that the true underlying effect size was consistent across all the included studies, which were drawn from similar populations. The highest reported rate of poor heart failure treatment outcomes was 32.6% in Amhara (6), while the lowest was 10.2% in Oromia (24) (Figure 2).

### Publication bias

**Figure 3:**
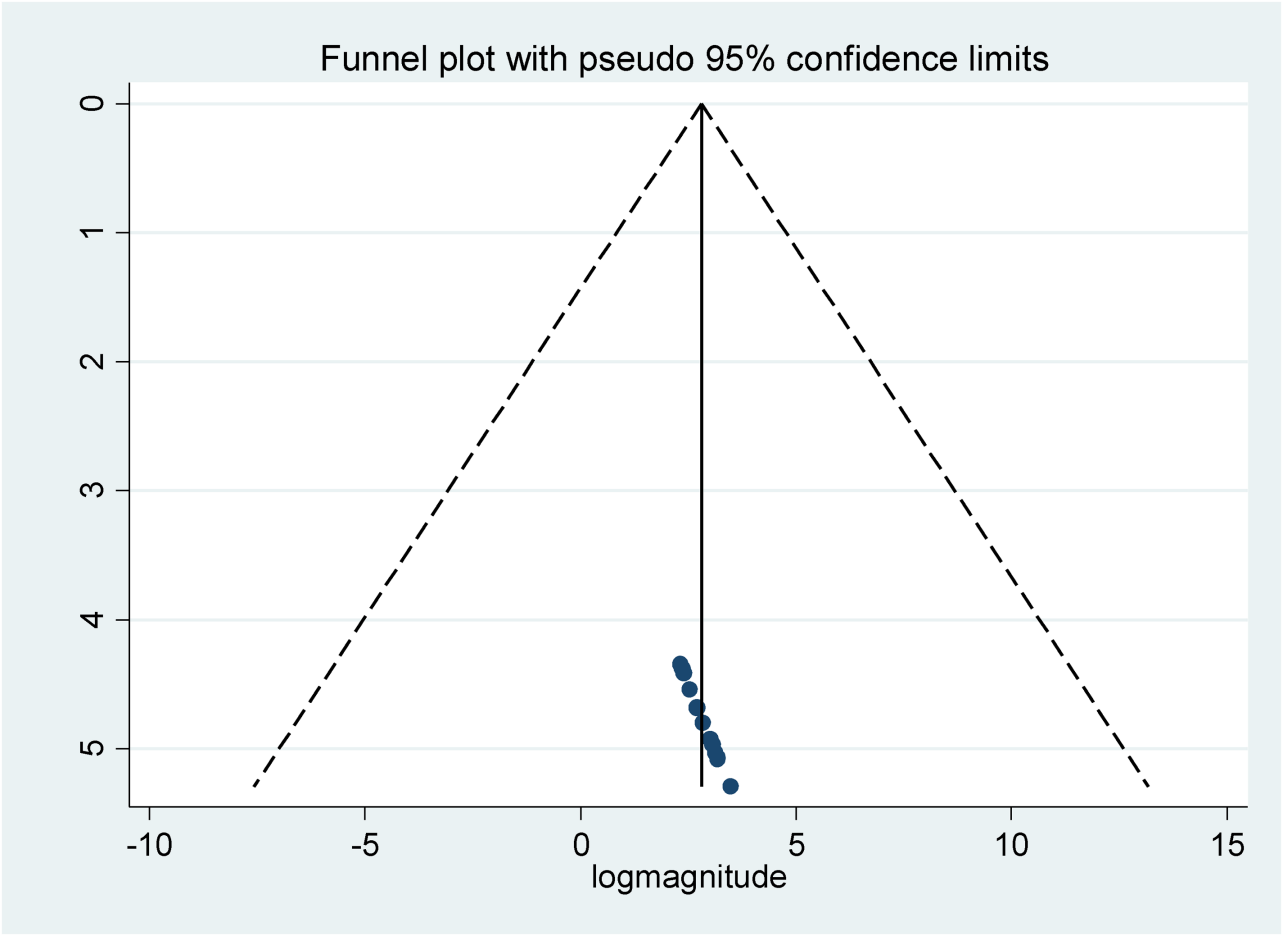
Funnel plot to test for publication bias in the included studies conducted in Ethiopia, 2024.

To assess the presence of publication bias, a funnel plot, Egger's test, and Begg's test were conducted at a 5% significance level. The funnel plot appeared symmetrical, and both the Egger test and Begg's test indicated no statistically significant evidence of publication bias, with *p*-values of 0.539 and 0.174, respectively (Figure 3).

### Factors associated with poor heart failure treatment outcomes

**Figure 4:**
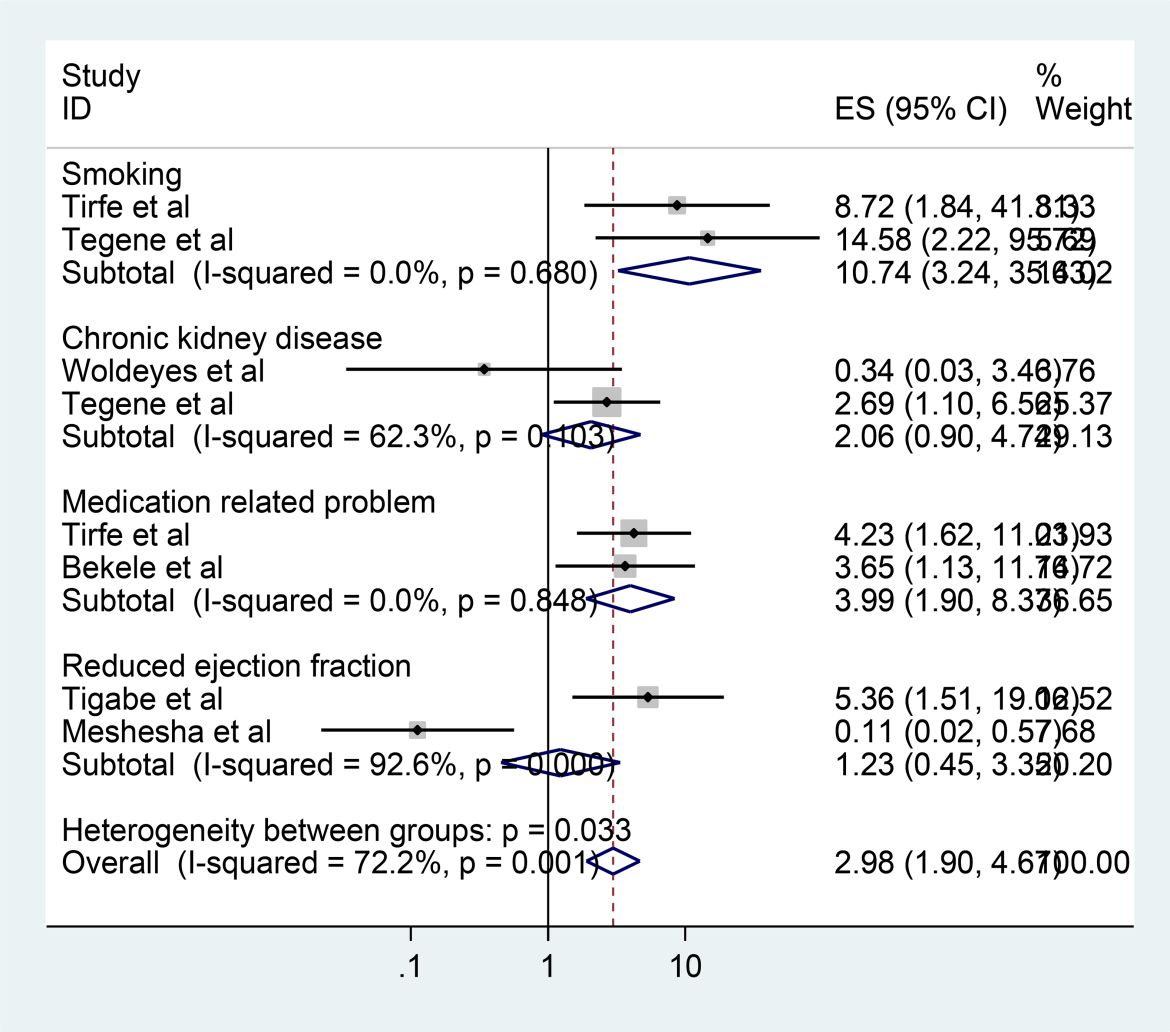
Forest plot of the factors associated with poor heart failure treatment outcomes in Ethiopia, 2024.

Six studies were included in the meta-analysis to identify pooled predictors of poor heart failure treatment outcomes. The pooled effect of the odds ratios was assessed using the command “metanlogor selogor, xlab(0.1,1,10) label(namevar=authors) by (factors) random texts(180)eform.” Patients with HF who smoked cigarettes were found to be 10.74 times more likely to experience poor treatment outcomes compared to their non-smoking counterparts [adjusted odds ratio (AOR) = 10.74; 95% CI, 3.24–35.63]. Similarly, patients who experienced medication-related problems were four times more likely to develop poor treatment outcomes than those without such problems (AOR = 3.99; 95% CI, 1.90–8.37) (Figure 4).

## Discussion

HF has been recognized as a significant contributor to the cardiovascular disease burden in sub-Saharan Africa for many decades (29). Therefore, our study aimed to assess the predictors of poor HF treatment outcomes in Ethiopia.

The pooled prevalence of poor HF treatment outcomes in Ethiopia was 16.67%, which was higher than studies conducted in Kenya (30) and Uganda (31). Compared to these previous studies, we found a higher incidence of in-hospital mortality. This may be attributable to the fact that these studies were carried out in highly specialized cardiac centers with superior diagnostic, therapeutic, and human resources, resulting in lower in-hospital mortality rates. In contrast, the current study was performed in the general medicine ward of a regional hospital, which reflects the level of care where the majority of patients with HF seek treatment in low-income countries such as Ethiopia.

The burden of HF was lower than that reported in studies conducted in Tanzania (6), Angola (32), and Burkina Faso (15). The variability in HF burden might be due to differences in patient characteristics, study designs, settings, duration, and the etiology of HF.

Patients with HF who smoked cigarettes were 10.74 times more likely to experience poor treatment outcomes compared to their counterparts. This finding aligns with studies conducted in Uganda and Tikur Anbessa Specialized Hospital in Addis Ababa, Ethiopia (17, 31). This correlation may be due to the cardiovascular risks associated with nicotine, which adversely affects the heart vessels and valves of tobacco smokers.

Patients with heart failure who had medication-related problems were 3.99 times more likely to have poor treatment outcomes than those who did not experience such problems. This finding is consistent with a study conducted in Angola, where medication non-compliance was identified as a precipitating factor of decompensated HF (32). Similar reports were noted in the study conducted in Tikur Anbessa Specialized Hospital (17). These issues may stem from the negative effects of drug therapy problems when attempting to optimize HF management. Drug-related problems (DRPs) are events involving medications that can impact a patient's desired therapeutic goals (33, 34).

The absence of clinical pharmacy services in Ethiopia may contribute to the prevalence of medication-related problems that adversely affect the treatment outcomes of patients with HF. In addition, insufficient interventions to reduce cigarette smoking persist, as smoking is often viewed as a social norm in Ethiopia. This cultural perception may contribute significantly to HF-associated mortality. Implementing pharmaceutical care services, including involving clinical pharmacists in patient rounds and establishing drug information services to address adverse drug effects, is essential. Furthermore, incorporating pharmacist assessment sheets into patient charts for evaluation and follow-up of drug therapy would be beneficial.

Interestingly, reduced ejection fraction was not identified as a predictor of HF mortality in our study. However, lower LVEF has been associated with poor HF treatment outcomes in Angola (32). This discrepancy may be related to differences in cut-off points for LVEF and the etiologies of HF across countries, which can influence mortality rates beyond just reduced ejection fraction. In our study, the three most common causes of HF were hypertensive heart disease, coronary artery disease, and dilated cardiomyopathy (25). In addition, patients with reduced ejection fraction often receive special attention in clinical practice, which may contribute to a lower mortality rate in Ethiopia.

The implications of these findings underscore the need for a multifaceted approach to managing HF in Ethiopia. Addressing the social determinants of health, such as smoking cessation and access to medications, is crucial for improving patient outcomes. Public health campaigns that aim to educate the community about the risks of smoking and the importance of medication adherence could significantly improve HF management. Moreover, enhancing healthcare infrastructure to improve access to specialized care and training healthcare providers could lead to better treatment adherence and outcomes.

This review calls for both governmental and non-governmental interventions to reduce mortality associated with HF. The study aims to assist policymakers by providing evidence to assist in the planning of various interventions specific to decreasing mortality from HF. Moreover, it will aid the government in developing standard treatment guidelines and evaluating the effectiveness of their programs for HF patients. This review will also guide clinical practice by providing appropriate treatments and addressing factors associated with poor HF outcomes, such as mortality. Finally, our study will serve as a benchmark for future researchers conducting interventional studies aimed at reducing HF hospitalization and mortality.

This systematic review and meta-analysis has several limitations that should be acknowledged. First, the included studies varied in design, sample size, and methodology, which may affect the generalizability of the findings. For instance, many studies were conducted in specific regions or hospitals, potentially limiting the applicability of the results to the broader Ethiopian population. In addition, the reliance on observational studies may introduce biases related to data collection and reporting, particularly regarding self-reported measures of smoking and medication adherence.

Moreover, the quality of the included studies varied, as indicated by the quality assessment. While most studies were deemed high quality, some were assessed as moderate quality, which may influence the robustness of the pooled estimates. Furthermore, the potential for publication bias exists as studies with negative results may be less likely to be published. This could skew the overall prevalence and predictors of poor HF treatment outcomes. Finally, the cross-sectional nature of some of the studies limited the ability to establish causal relationships between the identified predictors and treatment outcomes, suggesting that further longitudinal research is needed to clarify these associations.

## Conclusion

In this systematic review and meta-analysis, the prevalence of poor HF treatment outcomes in Ethiopia was found to be concerningly high. This study identified that smoking cigarettes and the presence of medication-related problems were significantly associated with adverse HF treatment outcomes. To address these critical issues, it is essential to implement educational interventions through community outreach programs aimed at raising awareness about the detrimental impact of smoking on mortality among patients with HF. In addition, healthcare workers should receive training focused on rational drug use to enhance treatment adherence and optimize patient outcomes. Furthermore, establishing pharmaceutical care services in Ethiopia is vital to effectively manage the adverse effects of medications and improve the overall care for patients with HF. By addressing these factors, we can significantly improve treatment outcomes and reduce mortality associated with HF in the region.

## Data Availability

The original contributions presented in the study are included in the article/Supplementary Material, further inquiries can be directed to the corresponding author.
